# Rho Kinase (ROCK) Inhibitors in the Treatment of Glaucoma and Glaucoma Surgery: A Systematic Review of Early to Late Phase Clinical Trials

**DOI:** 10.3390/ph18040523

**Published:** 2025-04-03

**Authors:** Jit Kai Tan, Peng Tee Khaw, Christin Henein

**Affiliations:** 1 Guy’s Campus, King’s College London, London SE1 1UL, UK; 2 Institute of Ophthalmology, University College London, London EC1V 9EL, UK; 3 National Institute for Health and Care Research Biomedical Research Centre for Ophthalmology, Moorfields Eye Hospital and University College London Institute of Ophthalmology, London EC1V 2PD, UK

**Keywords:** glaucoma, primary open-angle glaucoma, ocular hypertension, rho kinase inhibitors, glaucoma filtration surgery, fibrosis

## Abstract

**Background/Objectives**: Primary open-angle glaucoma (POAG) is an anterior optic neuropathy that can lead to irreversible vision loss if untreated. Prostaglandin analogues are the first-line treatment, but new drug classes, such as rho kinase (ROCK) inhibitors, are being explored. This review evaluates the efficacy and safety of ROCK inhibitors in treating POAG based on completed trials, comparing results with available natural history data and identifying areas for further research. **Methods**: A systematic database search was conducted in Ovid MEDLINE and Ovid Embase on 5 April 2022 using the following keywords: ‘glaucoma’, ‘rho kinase inhibitor’, ‘rho-kinase inhibitor’, ‘rock inhibitor’, ‘ripasudil’, ‘netarsudil’, and ‘fasudil’. Abstracts were screened for relevant studies and results summarized in tables. **Results**: The analysis of trials conducted for ROCK inhibitors reveals that they are a safe and efficacious drug to treat POAG, demonstrating non-inferiority to existing medical treatments. Comparison of data to natural history studies was inconclusive due to the lack of natural history studies and their limitations. The results showed ROCK inhibitors to be effective when combined with existing medical treatments. However, questions remain regarding the optimal dosage, patient selection, and cost-effectiveness. Outcome measures for future trials should be expanded to include additional methods of monitoring disease progression as well as patient quality-of-life. **Conclusions**: ROCK inhibitors have emerged with a favorable safety profile, efficaciously attenuating intraocular pressure. To elucidate their long-term therapeutic value and safety comprehensively, further independent, large-scale, prospective randomized controlled trials are warranted. Such studies are pivotal to augment our understanding of this emergent medication class.

## 1. Introduction

### 1.1. Glaucoma

In 2020, glaucoma affected over 75 million people worldwide, and projections suggest this number will escalate to 110 million by 2040. It remains the leading cause of progressive and irreversible blindness globally, with certain ethnicities displaying a predisposition to specific glaucoma types [1,2]. Glaucoma is the term used to describe a group of eye diseases that cause progressive optic neuropathy, and in which intraocular pressure (IOP) is a key modifiable factor. Glaucoma is commonly associated with raised IOP and is characterized by visual field defects and changes to the optic nerve head such as pathological cupping or, as a late sign, pallor of the optic disc. Ocular hypertension is where there is consistently or recurrently elevated IOP (greater than 21 mmHg) but with no signs of glaucoma [3].

This review examines the role of rho kinase (ROCK) inhibitors to lower the pressure in ocular hypertension (OHT) and glaucoma and in order to reduce progressive optic nerve damage and maintain visual function. We highlight their potential impact on ocular perfusion, neuroprotection, and anti-fibrotic properties after glaucoma filtration surgery. Moreover, this review critically evaluates the randomized controlled trials (RCT) conducted for this emergent class of medications.

#### 1.1.1. Anatomy and Physiology

**Figure 1:**
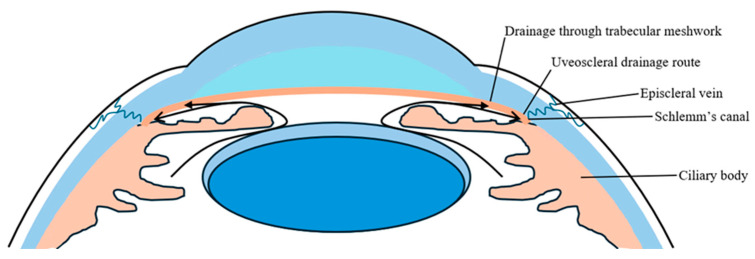
Image showing outflow route of aqueous humor in a healthy eye. Adapted from [6].

The aqueous humor is a transparent fluid that circulates through the anterior chamber of the eye, delivering nutrients to and eliminating metabolic waste from avascular ocular tissues such as the trabecular meshwork (TM). Synthesized by the ciliary body, this fluid is predominantly drained via the TM and the uveoscleral pathway located within the iridocorneal angle [4,5]. Structurally, the TM comprises three distinct regions: the uveal meshwork, the corneoscleral meshwork, and the juxtacanalicular tissue. These segments collectively constitute the principal resistance to aqueous outflow, working in concert with Schlemm’s canal to regulate intraocular pressure [5]. The aqueous humor is secreted by the ciliary body into the posterior chamber, then flows through the pupil into the anterior chamber, and finally the majority drains via the TM at the iridocorneal angle (Figure 1).

#### 1.1.2. Primary Open-Angle Glaucoma

Primary open-angle glaucoma (POAG) is the most prevalent form of open-angle glaucoma characterized by optic nerve damage due to elevated intraocular pressure (IOP), despite an open iridocorneal angle facilitating aqueous humor drainage from the eye’s anterior chamber. Accounting for over 70% of glaucoma cases globally, the etiology of POAG is multifaceted [7]. Significant risk factors include advanced age, heightened IOP, Black ethnicity, and a familial predisposition to the disease [8,9]. Several genetic markers, such as mutations in the myocilin and optineurin genes, have been linked to an increased susceptibility to POAG, with numerous additional associations uncovered through genome-wide association studies [10,11,12,13,14,15]. The pathogenetic mechanisms of POAG are complex, with existing genetic–environmental interactions making some individuals more at risk of developing glaucoma.

#### 1.1.3. Pathogenesis of POAG

Glaucoma represents a spectrum of ocular conditions that, through various mechanisms, damage the optic nerve [16]. Elevated IOP is widely recognized as the major modifiable factor in the pathogenesis of POAG, believed to compromise the integrity of the lamina cribrosa, consequently impairing retinal ganglion cells (RGC), their axons, and axoplasmic transport [17]. Damage to the lamina cribrosa and the axons with their support glial cells interrupts the neurotrophic support relayed from brain target neurons to RGCs, as well as neurometabolic support from the local glial cells, potentially initiating cellular apoptosis [18,19]. These events precipitate the degeneration of RGCs, creating a deleterious milieu mediated by mechanisms such as glutamate excitotoxicity, which further exacerbates RGC loss [19,20]. The ensuing, currently irreversible damage to the optic nerve manifests as visual field deficits, significantly compromising vision.

Additionally, the pathogenesis of POAG is thought to involve a myriad of other elements including autoimmune responses, structural vulnerabilities of the lamina cribrosa, mitochondrial dysfunction, and diminished ocular blood flow stemming from possible vascular compression and reduced intracranial pressure [21,22,23,24]. Although these factors are implicated, their roles are less understood, with very limited techniques of investigation. Moreover, excessive activation of the glutaminergic system, oxidative stress with resultant free radical formation, inflammatory cytokines, and immune dysregulation are also possible contributing factors to the disease process [16].

#### 1.1.4. Diagnosis

In POAG, pain is not a feature, and visual field defects are often detected at an advanced stage of damage, particularly where healthcare services are less well developed. Early diagnosis of POAG is crucial for preventing irreversible optic nerve damage, as symptoms become apparent only in the late stage of the disease. Diagnostic methods to detect POAG include direct ophthalmoscopy, gonioscopy, tonometry, and currently automated perimetry, with no single test being definitive in isolation [2,6]. Retinal and optic nerve imaging and in particular spectral-domain optical coherence tomography (SD-OCT), is now central to the diagnostic process, providing detailed assessments of the optic nerve and retinal nerve fiber layer (RNFL) [25,26].

### 1.2. The Natural History of POAG

The Early Manifest Glaucoma Trial examined progression of POAG in 46 patients with early glaucoma (including chronic simple glaucoma, normal-tension glaucoma, and exfoliative glaucoma) [27] and used the mean deviation index to determine the rate of visual field loss. The results found that patients with POAG was −1.08 dB per year, with a normal eye having 0 dB and a blind glaucoma eye having a range of −25–30 dB, giving approximately 25 years to progress from a normal eye to blindness. At the end of the study, 74% of patients showed progression in visual field loss, measured by a significant loss in light sensitivity in three consecutive tests at the same three or more test point locations [28].

A historical cohort study conducted in St. Lucia studied 287 eyes of 155 subjects and noted that 153 untreated eyes had worsened in visual field scores over one year according to the Advanced Glaucoma Intervention Study (AGIS) scoring system. The majority of these eyes were classified as having mild-moderate glaucoma. There was a significant association with increasing age, higher IOP, and progression of visual field defects [29].

#### 1.2.1. Medical Treatments for POAG

There are currently six classes of conventional medical treatments for POAG, which are prostaglandin analogues; beta blockers; carbonic anhydrase inhibitors; alpha agonists; parasympathomimetics; and more recently, rho kinase (ROCK) inhibitors. These drug classes act in different ways, ranging from increasing aqueous outflow to decreasing aqueous humor production. Over the past decade, there have been changing trends in prescribing practices with an increasing use of preservative-free preparations and combination therapies, with the aim to improve patient compliance through a simpler treatment regimen.

Prostaglandin analogues (PGA) such as latanoprost are the first-line medical treatment and act through increasing uveoscleral outflow and therefore lower IOP. Side effects of this medication include eyelash elongation, periocular pigmentation and conjunctival hyperemia, iris cysts, cystoid macular oedema, and peri-orbital fat atrophy [2,30]. Beta blockers (BB) such as timolol are usually added on to medical therapy if IOP is uncontrolled with a prostaglandin analogue alone, and they work through decreasing aqueous humor production [2]. Whilst the ocular side effect profile is low, there can be serious systemic side effects such as bronchospasm, hypotension, impotence, and insomnia [31].

Carbonic anhydrase inhibitors such as acetazolamide rapidly lower aqueous humor production through decreasing bicarbonate secretion into ciliary epithelial cells [32]. Side effects can range from mild, such as stinging and metallic taste in the mouth, to serious electrolyte imbalances and Stevens–Johnson syndrome [30]. Alpha agonists include brimonidine and work to lower IOP by both lowering aqueous production and raising uveoscleral outflow by influencing the sympathetic tone of the ciliary process [30]. It is important to note that this class of medication crosses the blood–brain barrier and can lead to central nervous system and respiratory depression [32]. The final class of drugs used in conventional medical treatment are parasympathomimetics such as pilocarpine that increase aqueous outflow through the TM [32]. Side effects of parasympathomimetics include miosis, brow aches, accommodative spasm, and induced myopia [33].

#### 1.2.2. Surgical Treatments for POAG

There are a variety of surgical treatments that are currently used to treat POAG, ranging from external aqueous filtering operations such as trabeculectomy to minimally invasive glaucoma surgery (MIGS). Trabeculectomies are the gold-standard surgical treatment for POAG, usually reserved in cases where IOP is insufficiently controlled by medication alone. Formation of a filtering bleb allows aqueous humor to flow from the anterior chamber to subconjunctival space, lowering IOP. However, subconjunctival fibrosis remains the leading cause of bleb failure and is the biggest challenge to successful filtration surgery [34]. Medical management tends to be used until it is definitively managed surgically. There is a growing use of preservative-free preparations of eyedrops in patients that are likely to require filtration surgery and improve bleb survival rate [35]. MIGS procedures tend to be indicated for mild to moderate glaucoma, with the aim of reducing medication burden, as well as the need for filtration surgery. New innovations are implantable devices that have sustained release of ocular antihypertensive agents such as travoprost [36].

### 1.3. Approved ROCK Inhibitors

**Figure 2:**
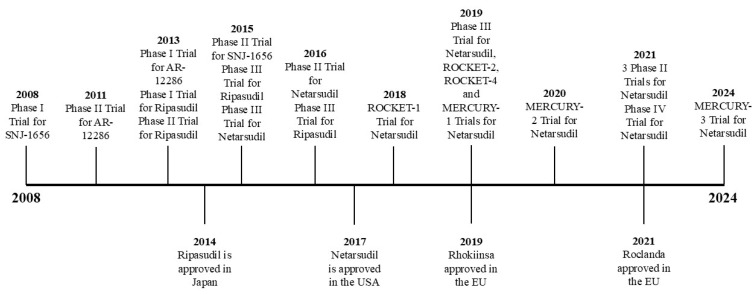
Timeline of ROCK inhibitor trials and years they were approved.

**Figure 3:**
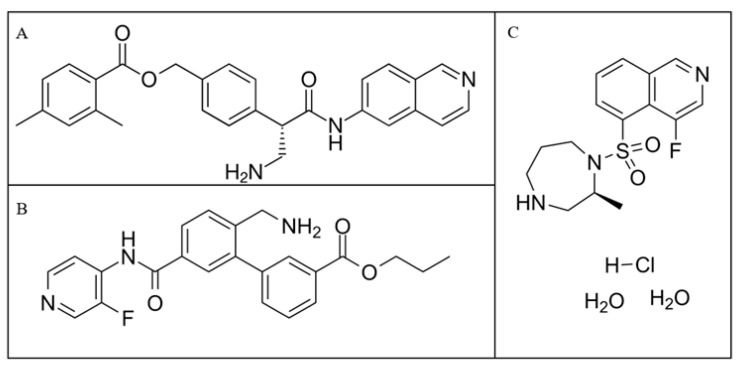
Chemical structures of (**A**) netarsudil, (**B**) sovesudil, (**C**) ripasudil. Netarsudil has the molecular formula C_28_H_27_N_3_O_3_ and weighs 453.5 g/mol; ripasudil has the molecular formula C_15_H_18_FN_3_O_2_S and weighs 323.4 g/mol; sovesudil has the molecular formula C_23_H_22_FN_3_O_3_ and weighs 407.4 g/mol.

ROCK inhibitors represent an innovative category of ocular hypotensive agents and are the first new class of anti-glaucoma medications to receive approval from the U.S. Food and Drug Administration (FDA) since latanoprost in the 1990s, indicated for the treatment of POAG and OHT [37]. Ripasudil (K-115) emerged as the inaugural ROCK inhibitor sanctioned for POAG treatment in Japan in 2014 (Figure 2). Subsequently, Rhopressa, which contains netarsudil (AR-13324), was granted FDA approval in the United States in 2017 for POAG management [38]. Additionally, Rhokiinsa, another formulation with netarsudil, received authorization in the European Union in 2019 for treating POAG and OHT [39]. Roclanda, a combination drop with netarsudil and latanoprost, has recently received approval in the European Union in 2021 for treatment of POAG and OHT [40]. Numerous other ROCK inhibitors are currently undergoing trials in both human and animal studies [41,42,43]. Chemical formulations of netarsudil, ripasudil, and sovesudil are shown in Figure 3.

#### 1.3.1. Mechanism of Action of ROCK Inhibitors

ROCK inhibitors function by impeding the activity of ROCK1 and ROCK2, which are the key effectors within the rho family of G-proteins, comprising RhoA, RhoB, and RhoC. These proteins are active when bound to guanosine triphosphate (GTP) and rendered inactive when bound to guanosine diphosphate [44,45]. The activation of these GTP-bound proteins leads to the stimulation of ROCK, which in turn influences a range of downstream effector tissues. Through this pathway, ROCK inhibitors can modulate various cellular functions, including cell adhesion, migration, smooth muscle contraction, proliferation, and the regulation of cell polarity [46,47,48,49].

#### 1.3.2. Increased Ocular Perfusion

Reduced blood flow to the optic nerve is linked to glaucomatous damage, with high IOP exacerbating optic nerve atrophy by disturbing perfusion and energy metabolism [50]. Additionally, low perfusion pressure and nocturnal blood pressure dips have been implicated in further optic nerve damage [51]. The ROCK pathway, integral to vascular tone regulation, is a therapeutic target to potentially improve ocular blood flow and mitigate vascular damage, as evidenced by animal studies showing increased blood flow with ROCK inhibitors [43,52,53,54]. Despite promising animal studies, the vasoprotective effects of ROCK inhibitors in humans remain to be confirmed, necessitating more comprehensive research to validate these findings and their clinical application.

#### 1.3.3. Neuroprotective Effects of ROCK Inhibitors

Raised IOP is believed to compress neuronal tissue at the lamina cribrosa, inhibiting growth of axons through inhibitory proteins such as myelin-associated glycoprotein. Neurometabolically supportive glial cells in the optic nerve head and the axons and cytoskeletal contraction may further this [18,19]. POAG upregulates the ROCK pathway, contributing to this inhibition, impacting cytoskeletal dynamics and cellular motility. ROCK inhibitors can counteract these effects, promoting axon regeneration and neuronal survival by influencing critical molecular pathways [51,55,56,57].

Animal studies have suggested that ROCK inhibitors reduce factors contributing to neuronal loss, such as reactive gliosis and leucocyte infiltration. Through these factors, axonal regeneration possibly following central nervous system injuries may be promoted by inhibition of the ROCK pathway [58,59,60,61,62,63]. As further research into the neuroprotective capabilities of ROCK inhibitors is conducted, caution must be exercised regarding their potential. Factors such as the limited regenerative capacity of adult neurons and the complexity of the neuronal growth environment must not be overlooked [64].

There is a paucity of trials combining ROCK inhibitors and brimonidine. Neuroprotective benefits of this combined agent are still unknown. Future studies should include optic nerve head thinning as an endpoint to assess the neuroprotective potential of this combination therapy. It would also be crucial to be stringent with the population recruited in terms of diet and any nutritional deficiencies which could reduce any neuroprotective effects.

#### 1.3.4. Cellular Effects of ROCK Inhibitors

Through inhibition of the rho family of G-proteins, ROCK inhibitors have a variety of effects on different cell types in the eye [61]. They have been reported to regulate cell morphology, adhesion, and apoptosis and alter IOP through modulation of the actin cytoskeleton [65,66,67,68]. Modification of ocular blood flow to the optic nerve head [53,69], reduction of scar formation, and inflammation following glaucoma surgery are also theorized effects of ROCK inhibitors [70,71].

Calcium-independent and -dependent pathways affect the trabecular meshwork. ROCK inhibitors only affect the calcium-independent pathway [54]. Activation of the ROCK pathway indirectly results in raised levels of actin filament density, thereby increasing stability and rigidity of the actin cytoskeleton [72]. Through these effector pathways, ROCK has demonstrated the ability to regulate cellular, migration, life cycle, and growth directly or indirectly through muscle cell contractility and the actin cytoskeleton [73].

**Figure 4:**
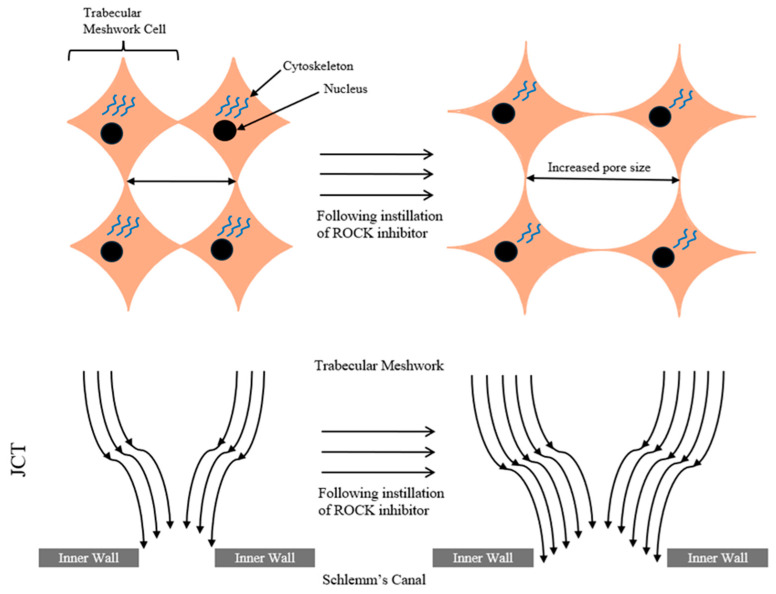
The ‘funneling’ effect seen in the TM.

Increased outflow resistance in SC is a downstream effect of this, reducing aqueous humor drainage in the juxtacanalicular connective tissue of SC, an effect called ‘funneling’ [37,74,75]. This effect is shown in Figure 4.

ROCK inhibitors have been shown to alter SC’s actin cytoskeleton through opposition of the ROCK pathway, reducing synthesis of the extracellular matrix, density of actin stress fibers, and cell tension and stiffness, easing and aiding aqueous humor outflow [37,76,77]. This is theorized to reduce the ‘funneling’ effect and outflow resistance, through greater pore formation in the juxtacanalicular region of SC from actin cytoskeleton modification [37].

ROCK inhibitors have a similar effect in the TM, due to the smooth-muscle-like properties of the TM and expression of α-smooth muscle actin [76]. ROCK inhibitors influence TM cells through the calcium-independent pathway [78], playing a critical role in myosin light chain (MLC) kinase and MLC phosphatase regulation [79]. ROCK inhibitor administration results in reduced levels of MLC phosphorylation in TM cells and tissue, lowering smooth muscle response to contractile agonists [76,80].

MLC is a regulator of myosin II, which can influence cell adhesions, shaping and initiating actin cross-bridging and the formation of cell-to-cell junctions when phosphorylated [79], raising resistance to aqueous humor outflow. ROCK inhibitors have been demonstrated to be able to reversibly oppose these changes and suppress prolonged contraction of actin stress fiber focal adhesions and TM cells facilitating aqueous humor outflow [80,81].

Inhibition of the ROCK pathway influences endothelin-1-induced ROCK signaling, in turn resulting in reduced vasoconstriction of retinal blood vessels and vasomotor tone through lowering smooth muscle contraction [82,83].

This increased perfusion to the optic nerve head, theorized to aid in glaucomatous disease [84]. ROCK inhibitors can also lower episcleral venous pressure [38], a contributing factor to increased IOP, as demonstrated by the modified Goldmann equation [85]. However, its significance in influencing IOP through this mechanism is not yet understood, as SC is the primary point of outflow resistance for aqueous humor.

### 1.4. Review Aims

This review aims to evaluate the safety and efficacy of ROCK inhibitors in the treatment of POAG, with a particular focus on their IOP-lowering effects based on clinical trial data. Additionally, it will compare the safety and efficacy profiles of ROCK inhibitors with existing medical treatments for POAG and explore their potential as adjunctive therapies. The review will also discuss current challenges in the field, including patient selection, optimal dosing strategies, and broader clinical applications, while identifying key areas for future research.

## 2. Results

**Figure 5:**
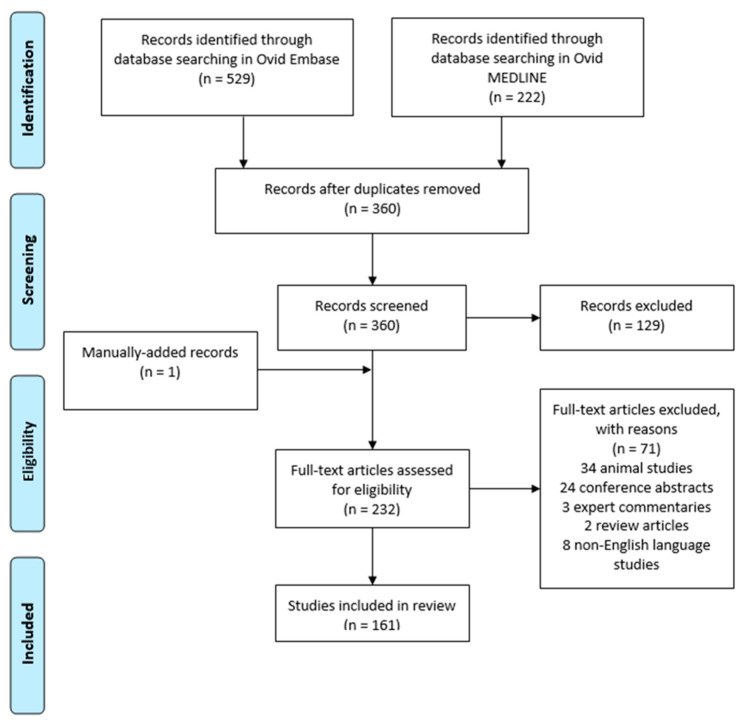
PRISMA diagram summarizing the steps for selection of studies [86].

Following searching three databases, 751 articles were identified for screening, and 232 full test articles were assessed (Figure 5). Of these, 161 met the eligibility criteria. One relevant article was manually added after reviewing a reference list. A total of 71 articles were excluded. Reasons for exclusion were 34 animal studies, 24 conference abstracts, 3 expert commentaries, 2 review articles, and 8 non-English language studies. No articles were excluded based on publication date. A total of 27 RCTs were identified, including 3 phase I trials, 8 phase II trials, 15 phase III trials, and 1 phase IV trial. Of these, 11 were conducted in Japan, 14 in the United States, and 1 in India, and 1 was a multicenter trial in the European Union. Two trials investigated SNJ-1656 (Senju Pharmaceutical Co. Ltd., Osaka, Japan), two examined AR-12286 (Aerie Pharmaceuticals, Inc., Durham, NC, USA), eight focused on ripasudil (Kowa Company, Ltd., Nagoya, Japan), and eleven evaluated netarsudil (Aerie Pharmaceuticals, Inc., Durham, NC, USA). Change in IOP from baseline was the primary endpoint in the majority of studies.

### 2.1. ROCK Inhibitor Trials

There have been numerous trials that look at ROCK inhibitors in the context of glaucoma, the first being SNJ-1656 in 2008 (Figure 2). In 2008, the first human trial that examined the safety and effectiveness of ROCK inhibitors in lowering IOP was the phase I trial for SNJ-1656, an ophthalmic solution of Y-39983, which is a selective ROCK inhibitor [87]. The trial was a randomized, double-masked, group-comparison study with a total of 45 patients divided into 5 groups that were instilled with SNJ-1656 in increasing doses (0.003%, 0.01%, 0.03%, 0.05%, 0.1%). After this trial was conducted, trials for netarsudil and ripasudil were conducted to evaluate the efficacy and safety of those drugs [88].

The first human trial for netarsudil was conducted in 2015 to compare efficacy with latanoprost and was a randomized, double-masked study conducted over 28 days with a total of 213 patients that completed the study [89]. Patients were randomized to receive netarsudil 0.01%, 0.02%, or 0.005% latanoprost. Table 1 summarizes the clinical trials that have been conducted to date. It should be noted that AR-12286 was abandoned by Aerie Pharmaceuticals in 2017 as netarsudil was judged to have a longer duration of action [44].

### 2.2. Patient Characteristics

The trials for ripasudil were focused on adults with a diagnosis of OHT or POAG [88]. The patients were excluded if they had any ocular comorbidities; undergone any ocular surgeries (other than cataract surgery more than 1 year ago, retinal laser treatment and yttrium–aluminum–garnet laser posterior capsulotomy more than 90 days ago, and eyelid surgery more than 120 days ago) [93,95]; severe visual field defects; impaired visual acuity; narrow iridocorneal angles; and any impairment to the liver, kidneys, heart, or endocrine system diseases. Baseline IOPs in the trials examined ranged from 13 to 35 mmHg. Baseline visual fields were not mentioned.

The trials for netarsudil similarly included adults with a diagnosis of OHT or POAG, with an IOP of >17 mmHg. Best corrected visual acuity was required to be at least 20/200 Snellen, following guidelines from the Early Treatment Diabetic Retinopathy Study. Patients were excluded if they had pseudo-exfoliation or pigment dispersion glaucoma, a history of angle closure or narrow iridocorneal angles, or previous glaucoma surgeries. Individuals with evidence of any ocular inflammation, clinically significant blepharitis, or conjunctivitis as well as women of childbearing potential who were pregnant, nursing, planning a pregnancy, or not using a medically acceptable form of birth control were excluded from these studies. Baseline IOPs in the trials examined ranged from 14 to 36 mmHg. Baseline visual fields were not mentioned.

### 2.3. Efficacy and Safety of ROCK Inhibitors

The most common adverse event reported was conjunctival hyperemia, which is a vasodilatory response of microvasculature in the conjunctiva due to increasing vascular smooth muscle relaxation from ROCK inhibitors [80]. The most serious adverse events in the trials examined included corneal erosion [95], visual field defects [96], conjunctival hemorrhage [89], reduced visual acuity [100], and non-infectious conjunctivitis [110]. However, these serious adverse events had a low incidence (<11.0%). A recent Cochrane review found that once-daily netarsudil 0.02% had an increased adverse event rate of 66 more events per 100 person-months when compared to placebo [112]. It also found that twice-daily ripasudil 0.4% + timolol 0.5% had a raised adverse event rate of 35 more events per 100 person-months compared to twice-daily 0.5% timolol monotherapy [112]. This is summarized in Table 2. It is important to note that most studies only reported conjunctival hyperemia; where other side effects have been mentioned, these are also included in the table. There has also been anecdotal evidence of bullous keratopathy following netarsudil use, which was reversed with steroid use and discontinuation of netarsudil [113].

### 2.4. Efficacy of ROCK Inhibitors

The primary efficacy endpoint across all but one study examined was IOP reduction from baseline measured either with a Goldmann or Perkins tonometer. A Cochrane review found that once-daily netarsudil 0.02% was more effective at reducing IOP by about 3.11 mmHg at <6 months when compared to placebo, and twice-daily ripasudil 0.4% + timolol 0.5% was more effective than twice-daily timolol 0.5% monotherapy by about 0.75 mmHg at <6 months [112].

#### 2.4.1. ROCK Inhibitors vs. Placebo

The first trial conducted for SNJ-1656 found significant changes in IOP changes between placebo and treatment eyes in the 0.03% and 0.1% solutions. The maximal change in IOP 4 h post-instillation of 0.1% SNJ-1656 was greater than all lower concentrations of SNJ-1656 [87]. Confidence intervals, baseline demographic characteristics, and individual patient results were not reported in this study. The first study for AR-12286 found that all three concentrations of the medication were statistically significant for reduction in IOP, with the peak effect resulting between 2 to 4 h after instillation. The largest IOP reduction was from the 0.25% AR-12286 formulation, given twice daily, up to −6.8 mmHg [92]. Again, confidence intervals and individual patient results were not reported.

A phase II study for sovesudil utilized three arms to investigate the efficacy of low-dose (0.25%) and high-dose (0.5%) sovesudil against a placebo over 4 weeks. The high-dose group demonstrated a statistically significant reduction in diurnal IOP at all timepoints compared to the placebo (*p* = 0.0146), although the difference between the low-dose and placebo groups was not significant (*p* = 0.1350) [114]. The phase I trial of ripasudil studied five groups of ten participants each, with eight participants receiving the treatment and two participants receiving a placebo once daily. The concentrations of ripasudil instilled were 0.05%, 0.1%, 0.2%, 0.4%, and 0.8%. The main findings of the trial were that repeated instillation did not increase IOP reduction for at least 7 days, and that twice-daily dosing at higher concentrations of ripasudil were clinically appropriate for use. Conjunctival hyperemia occurred in what appeared to be a dose-dependent manner, although severity and duration did not increase with repeated instillation [88]. In a phase II trial, 210 participants were divided randomly into four groups, to receive one of placebo, 0.1%, 0.2%, or 0.4% concentration of ripasudil to be instilled in both eyes twice daily. Significant differences in the adjusted mean of IOP change were found in all time points for the 0.1% and 0.4% concentration groups and at 2 h following instillation for the 0.2% group (*p* < 0.05) [93].

Another phase II trial that administered placebo, 0.2%, and 0.4% ripasudil to patients after a washout period between 5 and 30 days found that there were statistically significant differences in IOP reductions in both ripasudil dosages and placebo at all investigated timepoints except for the placebo at 24 h after first instillation (*p* < 0.05) [94]. One open-label phase III trial assessed long-term IOP reductions following trabeculectomy or trabeculectomy combined with cataract surgery with 3-month postoperative treatment with ripasudil. Peak reduction in IOP was found between baseline and month 12, which were −5.4 and −5.0 mmHg in the treatment and non-treatment groups, respectively. No statistically significant reductions in IOP were found throughout the follow-up period [97].

In the first phase II trial for netarsudil, it was found that netarsudil met the primary efficacy endpoint, reducing mean IOP by −3.5 mmHg, and the secondary efficacy endpoint of the study, which was a significant reduction in mean diurnal IOP change of −3.5 mmHg (*p* < 0.001) at the end of the study [104]. Another phase II trial found netarsudil 0.2% treatment resulted in an absolute change of −4.5 mmHg from baseline compared to an IOP change of −0.98 mmHg in the vehicle group, a statistically significant IOP reduction from baseline (*p* < 0.0001) [105]. A phase II trial found that mean reductions of IOP from baseline at the end of the study were −1.7, −4.1, −4.8, and −4.8 for the placebo, 0.01%, 0.02%, and 0.04%, respectively—a statistically significant difference in IOP reduction (*p* < 0.0001) at all concentrations. Time-matched analysis of change in IOP also found that each post-treatment time point in all netarsudil concentrations was lower than the placebo [99].

#### 2.4.2. ROCK Inhibitor Monotherapy vs. Other

A one-year phase III trial (Table 2) [95] enrolled 354 participants and divided them into four groups to receive either monotherapy of ripasudil 0.4%, additive therapy to PGA, additive therapy to BB, or fixed combination (FC) drugs. The peak mean IOP reductions at week 52 were −3.7 mmHg in the monotherapy group, −2.4 mmHg in the additive to PGA group, −3.0 mmHg in the additive to BB group, and −1.7 mmHg in the FC drug group. At week 52, monotherapy still yielded the highest mean IOP reduction when compared to troughs of all other treatment groups. Statistically significant differences in IOP reduction were found in all treatment groups at all timepoints (*p* < 0.01) [95].

In the first phase III trial for netarsudil (Table 2) [89], latanoprost 0.005% was used as a comparator ocular hypotensive drug against netarsudil 0.01% and 0.02%. At the endpoint of the study, mean diurnal IOP was reduced by 5.5, 5.7, and 6.8 mmHg in the netarsudil 0.01%, 0.02%, and latanoprost groups, respectively. Netarsudil failed to meet the criteria for non-inferiority to latanoprost, which was an upper 95% confidence limit of <1.5 mmHg for the difference between netarsudil and latanoprost. The main limitation of this study was that due to the relatively short study period, the long-term efficacy and safety profile of netarsudil could not be determined [89].

The ROCKET-1 study (Table 2) [100] was a phase III trial that compared efficacy of netarsudil 0.02% to timolol 0.5% in 411 participants. The mean changes in IOP from baseline ranged from −3.3 to −5.0 mmHg and −3.7 to −5.1 mmHg for netarsudil and timolol, respectively, which was significant at all treatment time points (*p* < 0.0001). However, netarsudil did not meet the criteria for non-inferiority to timolol [100]. The main limitation of the ROCKET-1 study is that half of the patients that were screened were on prostaglandin therapy [100].

The ROCKET-2 study (Table 2) [100] examined the safety and efficacy of netarsudil 0.02% and timolol 0.5% in 756 participants. Both treatment groups for netarsudil met the criteria for non-inferiority to timolol in this study, although both concentrations of netarsudil produced similar IOP reductions to timolol at all points of IOP measurement [100].

The ROCKET-4 study (Table 2) [106] compared the efficacy of once-daily netarsudil against twice-daily timolol in 708 participants for 6 months. Netarsudil met the criteria for non-inferiority to timolol, as the mean differences in IOP were within 1.5 mmHg at all timepoints of the study [106]. Only 186 patients from each treatment arm were included in the primary efficacy analysis, introducing the possibility of selection bias.

#### 2.4.3. ROCK Inhibitor Combination Therapy vs. Other

A phase III trial that compared the effects of combined ripasudil with either latanoprost or timolol to compare difference in efficacy of lowering IOP enrolled 413 participants that were divided into groups of 205 and 208 into ripasudil–latanoprost and ripasudil–timolol groups, respectively. The mean IOP reductions in the ripasudil–timolol study 2 h after instillation were −2.9 and −1.3 mmHg for the treatment and placebo groups, respectively. This represented a statistically significant difference in IOP reduction of −1.6 mmHg (95% CI, 1.1–2.1 mmHg) (*p* < 0.001). In the ripasudil–latanoprost study, the mean IOP reductions at baseline IOP were −3.2 and −1.8 mmHg for the treatment and placebo groups, respectively. This was a statistically significant difference of −1.4 mmHg (95% CI, 0.9–1.9 mmHg) (*p* < 0.001) [96]. Despite a significant difference during statistical analysis, the authors stated that they were not confident in the differences found in the ripasudil-latanoprost groups.

Another clinical trial observed that netarsudil, netarsudil/bimatoprost, and bimatoprost were all efficacious in IOP reduction. The mean IOP ranges for the netarsudil, netarsudil/bimatoprost, and bimatoprost groups were 17.5–18.6 mmHg (baseline 26.6 ± 4.8), 15.8–16.5 mmHg (from 24.6 ± 3.0), and 14.0–14.9 mmHg (baseline 24.6 ± 4.6), respectively, which were all statistically significant reductions in IOP (*p* < 0.001). Mean reductions in IOP from baseline were not provided [111].

A real-world trial examined netarsudil 0.02% therapy either as a replacement for existing medical therapies or as an addition to IOP-lowering medication. This study found that of the subgroups receiving netarsudil 0.02% monotherapy as a replacement for other IOP-lowering medication, the treatment-naïve subgroup (newly diagnosed previously untreated) had the greatest reduction in IOP by the end of study, which was −3.9 mmHg. The largest IOP reduction in the subgroups receiving netarsudil 0.02% as a complement to PGA therapy, lowering IOP by −5.5 mmHg [110].

In a 28-day trial of fixed-dose netarsudil–latanoprost (PG324) 0.01% and 0.02% against monotherapy of latanoprost 0.005% and netarsudil 0.02% for 292 participants, it was found that there were changes in IOP of −7.8, −8.6, −7.6, and −6.3 mmHg for PG324 0.01%, 0.02%, latanoprost, and netarsudil, respectively. At the end of the study, the group that received PG324 0.02% had 69% of patients with a mean diurnal IOP ≤ 18 mmHg, the highest percentage of participants out of all groups [102].

The MERCURY-1 trial examined the difference in efficacy between FC netarsudil/latanoprost netarsudil 0.02% monotherapy and latanoprost 0.005% monotherapy. Netarsudil/latanoprost also met the criteria for superiority to each individual medication at all nine points of measurement by lowering IOP by an additional 1.8–3.0 mmHg versus netarsudil and an additional 1.3–2.5 mmHg versus latanoprost [107]. The MERCURY-2 trial, which examined the same aims as the MERCURY-1 trial, found that netarsudil/latanoprost met the criteria for superiority to each active component at all timepoints measured by lowering IOP by an additional 2.2–3.3 mmHg versus netarsudil and an additional 1.5–2.4 mmHg versus latanoprost [108]. The limitations of the MERCURY-1 and -2 trials were that both trials were conducted for 3 months, making it challenging to fully characterize the safety and efficacy profile of netarsudil/latanoprost and provide insight as to how netarsudil/latanoprost compares with other FC products long-term or when used in conjunction with other ocular hypotensive drugs. Two-thirds of the patients enrolled across MERCURY-1 and -2 were undergoing prior ocular anti-hypotensive therapy, which could affect the results of the trials.

MERCURY-3 investigated the difference between netarsudil/latanoprost against bimatoprost/timolol for a total of 6 months. Netarsudil/latanoprost achieved non-inferiority to bimatoprost/timolol at all timepoints with a between-treatment IOP reduction of ≤1.5 mmHg through to month 3 of treatment (*p* < 0.05) [109]. MERCURY-3 had strict inclusion and exclusion criteria, which may have limited the variety of patients recruited, affecting the generalizability of the results to other populations.

#### 2.4.4. Adverse Events

Early trials showed researchers that the most common adverse event was conjunctival hyperemia, across all chemical formulations of ROCK inhibitors, which appeared to be largely self-limiting and remains the most common adverse event in the trials that follow. It is important to note that preservatives used in eye drops can also be a cause of conjunctival hyperemia. In summary, the conjunctival hyperemia rates for netarsudil and ripasudil monotherapy were 14.0–72.2% and 28.6–96.4%, respectively. The conjunctival hyperemia rates for netarsudil combination therapies were 30.7–83.3%. Conjunctival hyperemia rates for ripasudil monotherapy were not reported [95]. Comparatively, the rates of conjunctival hyperemia of PGA were 14.0–42.7%. It should be noted that some formulations of netarsudil and ripasudil contain benzalkonium chloride as a preservative [95,115], which is well-documented to cause ocular surface irritation [116].

In a trial examining the efficacy of sovesudil in normal tension glaucoma, a total of 43 patients out of 119 (36.1%) reported adverse events. Incidences of conjunctival hyperemia were 2.6%, 17.5%, and 24.4% in the placebo, low-dose, and high-dose sovesudil groups, respectively, with four subjects (9.8%) in the high-dose group and one subject (2.5%) in the low-dose group withdrawing from the study because of ocular adverse events that were unspecified [114].

Likewise, the most common adverse event in ripasudil trials was conjunctival hyperemia. Two phase III trials reported conjunctival hyperemia as the most common adverse event [94,95]. Three participants discontinued one of the trials solely due to conjunctival hyperemia. A total of 51 participants discontinued the trial due to blepharitis and/or allergic conjunctivitis, which resolved after discontinuation of ripasudil administration. Further analysis revealed that pollinosis-induced complications and monotherapy of ripasudil 0.4% were two significant factors related to patients discontinuing that study [95].

In a trial comparing combinations of ripasudil–latanoprost against ripasudil–timolol, the most common adverse event was conjunctival hyperemia (125/208). It should be noted that the addition of ripasudil to first-line drugs did not cause an increase in conjunctival hyperemia [96]. Another clinical study found that the most common adverse event following trabeculectomy or trabeculectomy combined with cataract surgery was hypotony in both treatment (7/38) and non-treatment groups (10/52). No cases of conjunctival hyperemia were reported in either group [97].

Conjunctival hyperemia was the most common adverse event in the trials for netarsudil. In the first phase III study conducted for netarsudil, 92 out of 224 participants reported conjunctival hyperemia. There were no changes of note to visual acuity or any other ocular or systemic measure [89]. Another trial found conjunctival hyperemia to be significantly increased in the netarsudil (31/43) and netarsudil/bimatoprost (26/32) groups (*p* < 0.001), with a higher severity than the bimatoprost monotherapy group (*p* < 0.001) [111].

In the ROCKET-1 and ROCKET-2 studies, the most common adverse event in the netarsudil treatment groups was conjunctival hyperemia (383/707). In the ROCKET-2 trial, it was noted that twice-daily instillation of netarsudil resulted in more frequent adverse events with moderate severity. Other common adverse events without visual sequalae in the ROCKET-1 and ROCKET-2 trials were corneal verticillata (70/707) and conjunctival hemorrhage (107/707); however, no netarsudil-related systemic issues were reported [101]. The ROCKET-4 trial saw a high incidence of conjunctival hyperemia in the treatment group compared to the control (47.9% vs. 9.2%), although it should be noted that some of these patients had conjunctival hyperemia at baseline [106].

The safety results of the MERCURY-1 and 2 studies were similar to those of the ROCKET trials, with conjunctival hyperemia being the most commonly reported adverse event, although it led to discontinuation of the study for 29 patients in the MERCURY-1 study [107,108]. MERCURY-3 also reported conjunctival hyperemia as the most common adverse event, followed by corneal verticillata. A total of 67 participants (30.7%) in the netarsudil/latanoprost and 19 participants (9.0%) in the bimatoprost/timolol group suffered from conjunctival hyperemia (*p* < 0.0001). An overall discontinuation rate in the study of 11.2% due to adverse events was observed, with 14 patients in the netarsudil/latanoprost group being discontinued due to conjunctival hyperemia [109]. Adverse events that occurred in the rest of trials include blurry vision, corneal deposits, increased lacrimation, reduced visual acuity, and conjunctival oedema [100,105,110].

### 2.5. Anti-Fibrotic Effects of ROCK Inhibitors After Glaucoma Filtration Surgery

Tissue growth factor beta (TGF-ß) signals trans-differentiation of conjunctival fibroblasts into myofibroblasts, focal cell adhesions, and cell contraction, and it is characterized by uncontrolled production, degradation of the extracellular matrix, and increased contractility [117].

Some studies have shown that administration of ROCK inhibitors following trabeculectomy can inhibit TGF-ß. This reduces expression of α-smooth muscle actin and lowers post-surgical scarring [76,117]. It was previously found that latanoprost, a first-line medication, induces collagen contraction, inhibiting post-surgical wound healing [118], but was suppressed by ROCK inhibitor administration [70].

Currently, there have not been any RCTs conducted examining the anti-fibrotic effect of ROCK inhibitors on trabeculectomy success, despite a previous study revealing no significant difference [119]. Muhlisa and colleagues observed the effects of ripasudil on trabeculectomy success in terms of reduction in post-operative procedures such as bleb needling. They reported that bleb needling was performed in 9/38 and 12/52 patients in the ripasudil-treated and non-ripasudil groups, respectively [97]. Should the anti-fibrotic effect of ROCK inhibitors prove significant, they could potentially serve a dual purpose in both reducing fibrosis and IOP.

### 2.6. The Economy of ROCK Inhibitors

A recent cost analysis based on the MERCURY-1 trial found that the costs per patient were USD 1673.61, USD 1654.73, and USD 1570.83 for netarsudil/latanoprost, netarsudil monotherapy, and latanoprost monotherapy, respectively, after 3 months of treatment in the United States. A cost effectiveness ratio of USD 46.17 per mmHg reduction in IOP was found, with netarsudil/latanoprost being the most cost-effective option for lowering IOP [120]. To our knowledge, this is the only cost analysis conducted for ROCK inhibitors. Although this study supports the implementation of ROCK inhibitors as a cost-effective treatment, further considerations such as the benefit in the best and worst seeing eye are required. For the UK, cost effectiveness should be expressed as incremental cost per quality-adjusted life year. Further consideration should be given to low-income countries with a high disease burden of POAG.

## 3. Discussion

### General Limitations of the Trials

The trials reviewed consistently measured IOP reduction but often neglected functional outcomes like OCT and visual field tests, which are pertinent to patients with POAG. Notably, all studies focused solely on patients with POAG or OHT. There was a lack of standardized reporting for adverse events, leading to inconsistent data across trials. None of the studies had a standardized method of adverse events reporting, such as the Consolidated Standards of Reporting Trials [121], which led to inconsistent reporting of adverse events. Throughout the studies reviewed, none of the studies conducted a direct comparison of different ROCK inhibitors, which is an area that warrants research.

Evidence suggests that ROCK inhibitors might offer neuroprotection and facilitate axonal repair, yet RCTs have not assessed their effects on structural or functional optic nerve head damage. The impact of ROCK inhibitors on post-surgical fibrosis and corneal endothelium remains unexplored in RCTs.

Ethnic diversity in trial participants was limited, with 66.7% (14/21) of RCTs reporting predominantly White cohorts. Previous studies have shown that ethnic minorities are heavily underrepresented in POAG trials [122]. As POAG disproportionately affects certain ethnic groups, it is crucial for future research to include a broader ethnic spectrum to assess differential drug responses.

Patience adherence is a vital point of consideration when assessing the clinical utility of this new class of medication. Side effects such as conjunctival hyperemia, while usually transient and self-resolving, can still play a role in negatively impacting adherence to treatment regimen [81]. However, a standardized tool to evaluate the impact of such factors on adherence remains to be seen. A lack of consensus on POAG-specific patient-reported outcome measures is still unclear, with 41 separate quality-of-life questionnaires identified, underscoring the need for a universally accepted method of measuring patient-reported outcome measures [123].

Conflicts of interest were prevalent, as most trial investigators had pharmaceutical affiliations, underscoring the importance of independent studies for unbiased results. These limitations are summarized in Table 3, using the Risk of Bias 2 Tool [124].

Although the trials demonstrated non-inferiority of IOP-lowering efficacy of ROCK inhibitors to conventional first-line medical treatments for POAG, it is still unclear whether ROCK inhibitors can exist as an option for monotherapy, with the quality of evidence rated low or very low by a recent Cochrane review [112]. It is clear from the studies that ROCK inhibitors can enhance IOP-lowering potential of conventional medications when combined, with a generally mild side effect profile that is usually transient. However, due to the chronic nature of POAG and the fact that many patients will remain on medical therapy in the long run, it is paramount to thoroughly investigate any long-term drug interactions between ROCK inhibitors and existing IOP-lowering therapy. Given the paucity of literature in this aspect due to the recent approval of ROCK inhibitors, the MERCURY trials are the only large RCTs that examined the efficacy and safety profile of ROCK inhibitors combined with conventional POAG medications. The MERCURY-2 trial was only conducted for 1 year, which is an inadequate amount of time in the context of a side-effect profile for a medication that is likely to be taken for decades. There are some studies that have designed a series of urea-based ROCK inhibitors to increase efficacy and lower adverse events, although more research on its toxicology is required [125].

There are a multitude of prospective and retrospective studies that examine the safety and efficacy profile of using ROCK inhibitors as an adjunctive therapy to first-line medications [126,127,128,129] and have found favorable results in efficacy and adverse events. However, because of their study design and smaller sample sizes, research of a higher level of evidence is required to fully assess the efficacy and safety profile of combining ROCK inhibitors and conventional treatment in the long term.

## 4. Conclusions

This review primarily focuses on adult patients with OHT and mild to moderate POAG with limited inclusion of other glaucoma subtypes. Despite extensive research on ROCK inhibitors, further insights could be gained through functional assessments (e.g., visual field testing, intraocular pressure measurements) and structural evaluations (e.g., spectral-domain optical coherence tomography for retinal nerve fiber layer thickness, angiography for ocular perfusion). Additionally, incorporating patient-reported outcomes on treatment adherence and acceptability would provide a more comprehensive understanding of the therapeutic impact.

ROCK inhibitors have been validated as effective and well-tolerated, with an overall favorable safety profile despite some reported serious adverse events. Selection and reporting biases, along with inconsistent adverse event reporting, are areas needing refinement in future trials. While the long-term role of ROCK inhibitors in clinical practice appears promising, further research is required to fully establish their therapeutic position, particularly in combination with existing treatment options.

### Future Directions

Significant progress has been made in understanding the therapeutic effects of ROCK inhibitors in lowering IOP. However, several challenges remain, and further research is needed to explore their full potential and determine additional clinical benefits. ROCK inhibitors have demonstrated IOP-lowering efficacy, but their impact on patients with varying severities of POAG remains unclear. It is hypothesized that these inhibitors may be more effective in the early stages of POAG due to their mechanism of action, which modifies the TM structure and function. In advanced POAG, irreversible TM damage and Schlemm’s canal collapse may limit their therapeutic utility [112,130]. However, the specific stage beyond which ROCK inhibitors lose effectiveness has yet to be determined.

Optimizing dosage and treatment regimens remains a key area for further research. While higher doses of ROCK inhibitors have been linked to greater IOP reduction, they are also associated with an increased risk of adverse events. Determining an optimal dosing strategy could help minimize cosmetically undesirable side effects while maintaining therapeutic efficacy. Additionally, the development of an intraocular drug-releasing implant could offer a viable alternative for patients who face challenges with self-administering eye drops, ensuring more consistent and sustained drug delivery.

## 5. Materials and Methods

The key terms ‘rho kinase inhibitor’, ‘ripasudil’, ‘netarsudil’, ‘fasudil’, and ‘glaucoma’ were used, along with MeSH terms and free-text searches, to identify relevant concepts. A comprehensive literature search was conducted on 20 May 2024, using the MEDLINE (1946–present) and EMBASE (1974–present) databases. One reviewer (J.K.T.) independently screened the titles and abstracts of the retrieved articles using EndNote Software (Version 20.6, Bld 17174). Full-text articles of relevant studies were then analyzed, along with citations from their reference lists. Only randomized controlled trials were included. Two reviewers (J.K.T., C.H.) independently extracted key data, including study title, year of publication, author(s), number of participants, study design, type of intervention, primary outcomes measured, and main results. Non-English-language articles were excluded. Clinical trials involving human participants investigating ROCK inhibitors were included. Additionally, animal studies were considered if they provided novel insights into the management of key conditions. Studies involving patients under the age of 18 were excluded. Search strategies are provided in Appendixe A and Appendixe B.

### Inclusion and Exclusion Criteria

Studies were included if they evaluated ROCK inhibitors as a treatment option and exclusively involved patients diagnosed with OHT or POAG. Trials involving patients with other forms of glaucoma or those under the age of 18 were excluded.

The main themes of this review are as follows:

- Safety and efficacy outcomes of rho kinase inhibitor trials.
- Comparison of rho kinase inhibitors with conventional treatments for primary open-angle glaucoma.
- Additional benefits of rho kinase inhibitors in glaucoma management, including neuroprotective and anti-fibrotic effects.

## Figures and Tables

**Table 1:** Summary of trials conducted examining the efficacy of ROCK inhibitors on POAG.

Study	Phase	Indication	Study Design	Drug	Study Size	Duration of Treatment
SNJ-1656						
Tanihara et al. [87]	I	Healthy volunteers	Randomized, double-masked, group-comparison, placebo–control	Placebo (OD) SNJ-1656 0.003% (OD) SNJ-1656 0.01% (OD) SNJ-1656 0.03% (OD) SNJ-1656 0.05% (OD) SNJ-1656 0.1% (OD)	45 patients	7 days followed by 7 days of repeated instillation
Inoue et al. [90]	II	POAG OHT	Randomized, double-masked, multicenter, placebo–control	Placebo (BD) SNJ-1656 0.03% (BD) SNJ-1656 0.05% (BD) SNJ-1656 0.1% (BD)	63 (initially 66 patients)	7 days
AR-12286						
Kopczynski et al. [91]	I	Healthy volunteers	Randomized, double-masked, crossover study	AR-12286 0.5% with 0.0065% BC (OD) AR-12286 0.5% with 0.015% BC (OD)	18 patients	2 periods of 8 days with a 7-day washout period in between
Williams et al. (NCT00902200) [92]	II	POAG OHT	Randomized, double-masked, multicenter, vehicle–control	Vehicle (OD for 14 days, then BD for 7 days) AR-12286 0.05% (OD for 14 days, then BD for 7 days) AR-12286 0.1% (OD for 14 days, then BD for 7 days) AR-12286 0.25% (OD for 14 days, then BD for 7 days)	89 patients	3 consecutive 7-day periods
Ripasudil						
Tanihara et al. [88]	I	Healthy volunteers	Randomized, double-masked, placebo–control, group comparison	Placebo (OD) Ripasudil 0.05% (OD) Ripasudil 0.1% (OD) Ripasudil 0.2% (OD) Ripasudil 0.4% (OD) Ripasudil 0.8% (OD)	50 patients	7 days followed by 7 days of repeated instillation
Tanihara et al. [93]	II	POAG OHT	Randomized, double-masked, multicenter, placebo–control, group comparison	Placebo (BD) Ripasudil 0.1% (BD) Ripasudil 0.2% (BD) Ripasudil 0.4% (BD)	210 patients	8 weeks
Tanihara et al. [94]	III	POAG OHT	Randomized, open label, Latin-square crossover	Placebo (BD) Ripasudil 0.2% (BD) Ripasudil 0.4% (BD)	28 (initially 43 patients)	3 periods of 2 days each
Tanihara et al. [95]	III	POAG OHT Exfoliation glaucoma Pigmentary glaucoma	Non-randomized, multicenter, open-label	Ripasudil 0.4% (BD) Ripasudil 0.4% with PGA (BD) Ripasudil 0.4% with BB (BD) Ripasudil 0.4% with FC PGA and BB (BD)	388 patients	52 weeks
Tanihara et al. (JAPIC111700) [96]	III	POAG OHT	Randomized, double-masked, multicenter, placebo–control	Placebo (BD) Ripasudil 0.4% (BD)	208 patients	8 weeks
Tanihara et al. (JAPIC111700) [96]	III	POAG OHT	Randomized, double-masked, multicenter, placebo–control	Placebo (BD) Ripasudil 0.4% (BD)	205 patients	8 weeks
Muhlisah et al. (UMIN000019017) [97]	III	POAG	Randomized, multicenter, open-label	Ripasudil (BD)	90 (initially 122 patients)	3 months
Tanihara et al. (Crossover Study) (jRCT2080225220) [98]	III	Healthy volunteers	Randomized, single-center, open-label	RBFC (BD) Ripasudil (BD) Brimonidine	18 patients	8 days
Netarsudil						
Bacharach et al. (NCT01731002) [89]	III	OAG OHT	Randomized, double-masked, multicenter	Latanoprost 0.005% (OD) Netarsudil 0.01% (OD) Netarsudil 0.02% (OD)	213 (initially 224 patients)	28 days
Aerie (NCT03310580) [99]	II	OAG OHT	Randomized, double-masked, multicenter	Netarsudil 0.02% (OD) Netarsudil 0.04% (OD)	42 patients	28 days
Serle et al. (NCT02207491) [100]	III	OAG OHT	Randomized, double-masked, multicenter	Timolol 0.5% (BD) Netarsudil 0.02% (OD)	411 patients	3 months
Kahook et al. (NCT02207621) [101]	III	OAG OHT	Randomized, double-masked, multicenter	Timolol 0.5% (BD) Netarsudil 0.02% (OD) Netarsudil 0.02% (BD)	756 patients	3-month data reported for 12-month trial
Lewis et al. (NCT02057575) [102]	II	OAG OHT	Randomized, double-masked, multicenter	Latanoprost 0.005% and Netarsudil 0.01% (OD) Latanoprost 0.005% and Netarsudil 0.02% (OD) Latanoprost 0.005% (OD) Netarsudil 0.02% (OD)	292 (initially 298 patients)	28 days
Aerie (NCT02246764) [103]	III	OAG OHT	Randomized, double-masked, multicenter	Netarsudil 0.02% and Placebo (OD) Netarsudil 0.02% (BD) Timolol 0.5% (BD)	83 patients	12 months
Peace et al. (NCT02874846) [104]	II	OAG OHT	Randomized, double-masked, vehicle-control	Vehicle (OD) Netarsudil 0.02% (OD)	12 patients	9 days
Sit et al. (NCT03233308) [105]	II	POAG OHT	Randomized, double-masked, vehicle–control	Vehicle (OD) Netarsudil 0.02% (OD)	40 patients	7 days
Araie et al. (NCT03844945) [99]	II	POAG OHT	Randomized, double-masked, parallel-group, placebo–control	Placebo (OD) Netarsudil 0.01% (OD) Netarsudil 0.02% (OD) Netarsudil 0.04% (OD)	207 (initially 215 patients)	4 weeks
Khouri et al. (NCT02558374) [106]	III	OAG OHT	Randomized, double-masked	Timolol 0.5% (BD) Netarsudil 0.02% (OD)	708 patients	6 months
Asrani et al. (NCT02558400) [107]	III	OAG OHT	Randomized, double-masked	Latanoprost 0.005%and Netarsudil 0.02% FC (OD) Latanoprost 0.005% (OD) Netarsudil 0.02% (OD)	718 patients	3-month data reported for 12-month trial
Walters et al. (NCT02674854) [108]	III	OAG OHT	Randomized, double-masked	Latanoprost 0.005% FC and Netarsudil 0.02% (OD) Latanoprost 0.005% (OD) Netarsudil 0.02% (OD)	750 patients	3 months
Stalmans et al. (NCT03284853) [109]	III	OAG OHT	Randomized, double-masked, parallel-group, multicenter, active-control	Latanoprost 0.005% and netarsudil 0.02% FC (OD) Bimatoprost 0.03% and timolol 0.5% FC (OD)	430 patients	6 months
Zaman et al. (NCT03808688) [110]	IV	OAG OHT	Prospective, interventional, open-label, multicenter	Netarsudil 0.02% (OD)	242 (initially 262 patients)	12 weeks
Shahid et al. [111]	III	OAG OHT	Randomized, parallel-group, open-label	Netarsudil 0.02% (OD) Netarsudil 0.02% and bimatoprost 0.01% (OD) Bimatoprost 0.01% (OD)	109 (initially 133 patients)	12 weeks

Abbreviations: OD = once-daily, POAG = primary open-angle glaucoma, OHT = ocular hypertension, BD = twice daily, BC = benzalkonium chloride, PGA = prostaglandin analogue, BB = beta blocker, FC = fixed combination, RBFC = ripasudil–brimonidine fixed-dose combination, OAG = open-angle glaucoma.

**Table 2:** Summary of efficacy and safety of ROCK inhibitor trials.

Study	Duration of Follow-Up	Baseline IOP Inclusion Range	Drug (Number of Participants)	Baseline IOP mmHg (SD)	Efficacy mmHg (SD)	Frequent Adverse Events
SNJ-1656						
Tanihara et al. [87]	7 days	-			Change in IOP from baseline after 2 h	Conjunctival hyperemia
			Placebo (15)	14.1 (2.5)	−0.9	0.0%
			SNJ-1656 0.003% (6)	14.1 (1.4)	−1.2	16.7%
			SNJ-1656 0.01% (6)	13.7 (1.5)	−1.5	0.0%
			SNJ-1656 0.03% (6)	13.7 (2.2)	−2.2	33.3%
			SNJ-1656 0.05% (6)	13.2 (1.4)	−1.5	83.3%
			SNJ-1656 0.1% (6)	13.4 (2.7)	−2.0	100.0%
Inoue et al. [90]	7 days	22 ≤ IOP ≤ 31 mmHg			Change in IOP from baseline at peak	Conjunctival hyperemia
			Placebo (16)	~22.5	−1.5 (2.2)	Not reported
			SNJ-1656 0.03% (15)		−5.0 (2.4)	60.0%
			SNJ-1656 0.05% (14)		−4.4 (2.7)	100.0%
			SNJ-1656 0.1% (18)		−4.5 (1.9)	83.0%
AR-12286						
Kopczynski et al. [91]	8 days	14 ≤ IOP ≤ 20 mmHg			Change in IOP from baseline at peak	Conjunctival hyperemia
			AR-12286 0.5% with 0.015% BC (9)	17.0 (2.3)	−7.2 (2.0)	6.0%
			AR-12286 0.5% with 0.0065% BC (9)		−6.9 (1.8)	0.0%
Williams et al. (NCT00902200) [92]	22 days	21 ≤ IOP ≤ 24 mmHg			Change in IOP from baseline at peak	Conjunctival hyperemia
			Vehicle (22)	26.3 (2.5)	−2.4	9.1%
			AR-12286 0.05% (22)	26.0 (2.2)	−4.1	27.3%
			AR-12286 0.1% (23)	27.3 (3.2)	−5.0	39.1%
			AR-12286 0.25% (22)	26.9 (2.0)	−6.0	59.1%
Ripasudil						
Tanihara et al. [88]	7 days	≥13 mmHg			Change in IOP from baseline after 2 h	Conjunctival hyperemia
			Placebo (9)	15.1 (2.3)	−1.6	0.0%
			0.05% Ripasudil (8)	14.3 (2.5)	−3.4	62.5%
			Ripasudil 0.1% (7)	13.1 (1.8)	−2.2	57.1%
			Ripasudil 0.2% (8)	14.5 (2.5)	−2.6	62.5%
			Ripasudil 0.4% (7)	13.9 (1.5)	−4.0	28.6%
			Ripasudil 0.8% (8)	13.1 (1.4)	−4.3	87.5%
Tanihara et al. [93]	8 weeks	21 < IOP < 35 mmHg			Change in IOP from baseline at peak	Conjunctival hyperemia
			Placebo (54)	23.0 (2.1)	−2.5	13%
			Ripasudil 0.1% (53)	23.3 (2.4)	−3.7	43%
			Ripasudil 0.2% (54)	23.2 (2.0)	−4.2	57%
			Ripasudil 0.4% (49)	23.2 (1.9)	−4.5	65%
Tanihara et al. [94]	2 days	21 < IOP < 30 mmHg			Change in IOP from baseline after 2 h	Conjunctival hyperemia
			Placebo (28)	22.0 (2.1)	−4.1	10.7%
			Ripasudil 0.2% (28)		−6.8	78.6%
			Ripasudil 0.4% (28)		−7.3	96.4%
Tanihara et al. [95]	52 weeks	15 < IOP < 35 mmHg			Change in IOP from baseline at peak	Conjunctival hyperemia (74.6%) Blepharitis (20.6%) Allergic conjunctivitis (17.2%)
			Ripasudil 0.4% (173)	19.3 (2.7)	−3.7	
			Ripasudil 0.4% + with PGA (62)	17.6 (2.0)	−2.4	
			Ripasudil 0.4% + BB (60)	18.2 (2.3)	−2.0	
			Ripasudil 0.4% + FC PGA + BB (59)	17.6 (2.0)	−1.7	
Tanihara et al. (JAPIC111700) [96]	8 weeks	IOP ≥ 18 mmHg on Timolol			Change in IOP from baseline at peak	Conjunctival hyperemia
			Placebo (104)	19.7 (1.7)	−1.3	5.8%
			Ripasudil 0.4% (104)	19.9 (1.9)	−2.9	65.4%
Tanihara et al. (JAPIC111701) [96]	8 weeks	IOP ≥ 18 mmHg on Latanoprost			Change in IOP from baseline at peak	Conjunctival hyperemia
			Placebo (103)	19.6 (1.9)	−1.8	8.7%
			Ripasudil 0.4% (102)	20.1 (1.9)	−3.2	55.9%
Muhlisah et al. (UMIN000019017) [97]	3 months	-			Change in IOP from baseline at end of study	Hypotony, choroidal detachment
			Control (52)	16.2 (4.4)	−5.3	19.2%, 3.8%
			Ripasudil (38)	16.8 (5.0)	−6.2	18.4%, 10.5%
Tanihara et al. (Crossover Trial) (jRCT2080225220) [98]	8 days	IOP > 15 mmHg			Change in IOP from baseline at end of study	Conjunctival hyperemia
			RBFC (6)	12.7 (2.4)	−3.7	100.0%
			Ripasudil (6)	Not recorded	Not recorded	100.0%
			Brimonidine (6)	Not recorded	Not recorded	58.8%
Netarsudil						
Bacharach et al. (NCT01731002) [89]	28 days	24 ≤ IOP ≤ 36 mmHg			Change in IOP from baseline after 28 days	Conjunctival hyperemia
			Netarsudil 0.01% (74)	25.8	−5.4	52.0%
			Netarsudil 0.02% (72)	25.6	−5.9	57.0%
			Latanoprost 0.005% (77)	25.5	−6.8	16.0%
Aerie (NCT03310580) [99]	28 days	15 ≤ IOP < 30 mmHg			Mean diurnal iop at 28 days	Conjunctival hyperemia
			Placebo (15)		17.3	0.0%
			Netarsudil 0.02% (15)		14.4	66.7%
			Netarsudil 0.04% (12)		14.3	71.4%
Serle et al. (NCT02207491) [100]	3 months	17 < IOP < 27 mmHg			Change in IOP from baseline after 3 months (diurnal range)	Conjunctival hyperemia, conjunctival hemorrhage
			Netarsudil 0.02% (202)	22.5	−3.3 to −5.0	53.0%, 13.0%
			Timolol 0.5% (209)	22.3	−3.7 to −5.1	7.0%, 0.5%
Kahook et al. (NCT02207621) [101]	3 months	17 < IOP < 27 mmHg			Change in IOP from baseline after 3 months (diurnal range)	Conjunctival hyperemia, conjunctival hemorrhage, corneal verticillata
			Netarsudil 0.02% (251)	21.4	−3.3 to −4.6	50.0%, 15.0%, 9.0%
			Netarsudil 0.02% (254)	21.5	−4.1 to −5.4	59.0%, 17.0%, 15.0%
			Timolol 0.5% (251)	21.5	−3.7 to −5.1	10.0%, 0.0%, 0.4%
Lewis et al. (NCT02057575) [102]	28 days	24 ≤ IOP < 36 mmHg			Change in IOP from baseline after 28 days	Conjunctival hyperemia
			Latanoprost and netarsudil 0.01% (74)	25.1 (2.3)	−7.8	41.0%
			Latanoprost and netarsudil 0.02% (73)	25.1 (2.4)	−8.6	40.0%
			Latanoprost (73)	26.0 (2.8)	−7.6	14.0%
			Netarsudil 0.02% (78)	25.4 (2.7)	−6.3	40.0%
Peace et al. (NCT02874846) [104]	9 days	17 < IOP < 30 mmHg			Change in IOP from nocturnal baseline	None
			Vehicle (4)	22.9 (1.3)	−3.5	
			Netarsudil 0.2% (8)	22.4 (2.1)	−0.4	
Sit et al. (NCT03233308) [105]	7 days	17 < IOP < 30 mmHg			Change in IOP from baseline after 7 days	Conjunctival hyperemia
			Vehicle (18)	23.0 (1.4)	−4.5	0.0%
			Netarsudil 0.2% (18)	22.9 (1.6)	−1.0	72.2%
Araie et al. (NCT03844945) [99]	4 weeks	14 < IOP < 30 mmHg			Change in IOP from baseline after 4 weeks	Conjunctival hyperemia
			Placebo (55)	21.1 (3.7)	−1.7 (1.8)	1.8%
			Netarsudil 0.01% (54)	20.5 (2.8)	−4.1 (2.1)	23.6%
			Netarsudil 0.02% (51)	20.3 (2.8)	−4.8 (1.8)	37.0%
			Netarsudil 0.04% (55)	20.8 (3.2)	−4.8 (2.2)	56.9%
Khouri et al. (NCT02558374) [106]	6 months	20 < IOP < 30 mmHg			Change in IOP from baseline after 6 months	Conjunctival hyperemia
			Netarsudil 0.02% OD (351)	22.4	−3.9 to −4.7	47.9%
			Timolol 0.5% BD (357)	22.4	−3.8 to −5.2	9.2%
Asrani et al. (NCT02558400) [107]	3 months	20 < IOP < 36 mmHg			Change in IOP from baseline after 3 months (mean diurnal)	Conjunctival hyperemia
			Latanoprost 0.005% and netarsudil 0.02% FC OD (238)	23.7	−8.1	53.4%
			Latanoprost 0.005% OD (243)	23.6	−5.5	41.0%
			Netarsudil 0.02% OD (237)	23.5	−6.4	14.0%
Walters et al. (NCT02674854) [108]	3 months	20 < IOP < 36 mmHg			Change in IOP from baseline after 3 months (mean diurnal)	Conjunctival hyperemia
			Latanoprost 0.005% and netarsudil 0.02% FC OD (245)	23.5	−7.6	54.5%
			Latanoprost 0.005% OD (255)	23.6	−5.0	42.7%
			Netarsudil 0.02% OD (250)	23.5	−6.0	22.3%
Stalmans et al. (NCT03284853) [109]	6 months	IOP ≥ 17 mmHg in at least one eye IOP < 28 mmHg in both eyes			Change in IOP from baseline after 3 months	Conjunctival hyperemia, cornea verticillata
			Latanoprost 0.005% and netarsudil 0.02% FC OD (218)	25.1 (3.4)	−9.9	30.7%, 11.0%
			Bimatoprost 0.03% and timolol 0.5% FC OD (212)	24.8 (3.3)	−10.4	9.0%, 0.0%
Zaman et al. (NCT03808688) [110]	12 weeks	-			Change in IOP from Baseline after 12 weeks	Conjunctival hyperemia
			Netarsudil 0.02% (24)	19.4 (4.5)	−3.9 (3.6)	22.2%
			Netarsudil 0.02% replacing PGA (57)		−0.6 (3.1)	
			Netarsudil 0.02% replacing 2 concomitant therapies (6)		0.0 (1.6)	
			Netarsudil 0.02% replacing non-PGA monotherapy (4)		0.5 (1.7)	
			Netarsudil 0.02% and PGA (55)	20.3 (5.0)	−5.5 (5.0)	19.9%
			Netarsudil 0.02% and BB (2)		−4.3 (2.8)	
			Netarsudil 0.02% replacing ≥ 1 classes of concomitant therapy (64)		−0.4 (2.5)	
			Netarsudil 0.02% and ≥ 2 classes of concomitant therapy (64)		−4.5 (4.1)	
Shahid et al. [111]	12 weeks	21 < IOP < 32 mmHg			Change in IOP from baseline after 12 weeks	Conjunctival hyperemia
			Netarsudil 0.02% (43)	26.6 (4.8)	-	71.4%
			Netarsudil 0.02% and bimatoprost 0.01% (32)	24.6 (3.0)	-	83.3%
			Bimatoprost 0.01% (34)	24.6 (4.6)	-	15.3%

Abbreviations: IOP = intraocular pressure, SD = standard deviation, BC = benzalkonium chloride, PGA = prostaglandin analogue, BB = beta blocker, FC = fixed combination, RBFC = ripasudil–brimonidine fixed-dose combination, OD = once daily, BD = twice daily.

**Table 3:** Summary of critical appraisal of trials included in this study following the Risk of Bias 2 Tool and conflict of interest declarations.

Study	Randomization Process	Deviation from Intended Interventions	Missing Outcome Data	Measurement of the Outcome	Selection of the Reported Results	Conflict of Interest	Overall
Tanihara et al. [87]	Low risk	Low risk	Low risk	Low risk	Low risk	High risk	Some concerns
Inoue et al. [90]	Low risk	Low risk	Low risk	Low risk	Some concerns	High risk	Some concerns
Williams et al. [92]	Low risk	Low risk	Low risk	Low risk	Low risk	High risk	Some concerns
Kopczynski et al. [91]	Low risk	Low risk	Low risk	Low risk	Low risk	High risk	Some concerns
Tanihara et al. [88]	Low risk	Low risk	Low risk	Low risk	Low risk	High risk	Some concerns
Tanihara et al. [93]	Low risk	Some concerns	Low risk	Low risk	Low risk	High risk	Some concerns
Tanihara et al. [94]	Low risk	Low risk	Low risk	Low risk	Low risk	High risk	Some concerns
Tanihara et al. [95]	High risk	Low risk	Low risk	Low risk	Low risk	High risk	Some concerns
Tanihara et al. [96]	Low risk	Low risk	Low risk	Low risk	Low risk	High risk	Some concerns
Tanihara et al. [96]	Low risk	Low risk	Low risk	Low risk	Low risk	High risk	Some concerns
Muhlisah et al. [97]	Low risk	Some concerns	Low risk	Low risk	Low risk	Low risk	Some concerns
Tanihara et al. (Crossover Study) [98]	Low risk	Low risk	Low risk	Low risk	Low risk	High risk	Some concerns
Bacharach et al. [89]	Some concerns	Low risk	Low risk	Low risk	Low risk	High risk	Some concerns
Serle et al. [100]	Low risk	High risk	High risk	Low risk	Low risk	High risk	High risk
Kahook et al. [101]	Low risk	High risk	High risk	Low risk	Low risk	High risk	High risk
Lewis et al. [102]	Low risk	Low risk	Low risk	Low risk	Low risk	High risk	Some concerns
Peace et al. [104]	Low risk	Low risk	Low risk	Low risk	Low risk	High risk	Some concerns
Sit et al. [105]	Low risk	Some concerns	Low risk	Low risk	Low risk	High risk	Some concerns
Araie et al. [99]	Low risk	Low risk	Low risk	Low risk	Low risk	High risk	Some concerns
Khouri et al. [106]	Low risk	High risk	High risk	Low risk	Low risk	High risk	High risk
Asrani et al. [107]	Low risk	Low risk	Low risk	Low risk	Low risk	High risk	Some concerns
Walters et al. [108]	Low risk	Low risk	Low risk	Low risk	Low risk	High risk	Some concerns
Stalmans et al. [109]	Some concerns	Low risk	Low risk	Low risk	Low risk	High risk	Some concerns
Shahid et al. [111]	Low risk	Low risk	Low risk	Low risk	Low risk	Low risk	Low risk
Zaman et al. [110]	High risk	Low risk	High risk	Low risk	Low risk	High risk	High risk

Low risk: The study is judged to be at low risk of bias for all domains for this result. Some concerns: The study is judged to raise some concerns in at least one domain for this result, but not to be at high risk of bias for any domain. High risk: The study is judged to be at high risk of bias in at least one domain for this result, or the study is judged to have some concerns for multiple domains in a way that substantially lowers confidence in the result.

## Data Availability

No new data were created or analyzed in this study. Data sharing is not applicable to this article.

## Appendix A

**Embase 1974–5 April 2022**		
**Search Lines**	**Search Terms**	**Search Results**
1	glaucoma *	100,842
2	exp glaucoma/	91,390
3	1 or 2	105,022
4	rho kinase inhibitor	4177
5	rho-kinase inhibitor	4177
6	rock inhibitor	2146
7	ripasudil *	219
8	netarsudil *	229
9	fasudil *	3030
10	5 or 6 or 7 or 8 or 9	7594
11	3 or 10	112,087
12	3 and 10	529
* Was the symbol used for truncated search terms		

## Appendix B

**Medline 1946–5 April 2022**		
**Search Lines**	**Search Terms**	**Search Results**
1	glaucoma *	77,322
2	exp glaucoma/	56,949
3	1 or 2	77,541
4	rho kinase inhibitor	1589
5	rho-kinase inhibitor	1589
6	rock inhibitor	1366
7	ripasudil *	131
8	netarsudil *	113
9	fasudil *	1374
10	5 or 6 or 7 or 8 or 9	3699
11	3 or 10	81,018
12	3 and 10	222
* Was the symbol used for truncated search terms		
